# A Case of Large Perianal Mucinous Adenocarcinoma Arising from Recurrent Abscess and Complex Fistulae

**DOI:** 10.1155/2020/1798543

**Published:** 2020-12-18

**Authors:** Fahmi Pramaditto Azmi, Nur Afdzillah Abdul Rahman, Luqman Mazlan, Normala Basiron, Farrah-Hani Imran

**Affiliations:** ^1^Colorectal Surgery Unit, Department of Surgery, UKM Medical Centre, Kuala Lumpur, Malaysia; ^2^Department of Plastic and Reconstructive Surgery, Hospital Kuala Lumpur, Malaysia; ^3^Plastic and Reconstructive Surgery Unit, Department of Surgery, UKM Medical Centre, Kuala Lumpur, Malaysia

## Abstract

Mucinous adenocarcinoma of the perianal region is an oncologic rarity posing a diagnostic and therapeutic dilemma for treating oncologists. This is due to the low number of reported cases, compounded by the lack of definitive therapeutic guidelines. It accounts for 2% to 3% of all gastrointestinal malignancies and is historically known to arise from chronic anal fistulas and ischiorectal or perianal abscesses. We hereby report an interesting case of perianal mucinous adenocarcinoma in a 66-year-old male initially treated for a horseshoe abscess with complex fistulae. He presented with a 6-month history of a discharging growth in perianal region and painful defecation associated with occasional blood mixed stools. An incisional biopsy from the ulcer revealed mucinous adenocarcinoma. Contrast-enhanced computed tomography (CT) scan and magnetic resonance imaging (MRI) scan showed a localized perianal growth which involves the internal and external sphincter as well as suspicious involvement in the posterior aspect of the levator ani/puborectalis sling, which was further confirmed with colonoscopy (see figures). With no preset treatment protocol for this rare entity, he was managed with an abdominoperineal resection (APR) and vertical rectus abdominis myocutaneous flap (VRAM) tissue reconstruction. He had a turbulent postoperative period including intestinal obstruction secondary to internal herniation of bowel resulting in flap failure. The subsequent perineal wound was managed conservatively with advanced wound care and has since completely healed.

## 1. Introduction

Mucinous cancer is a distinct form of colorectal cancer (CRC) found in 10–15% of patients with CRC. Mucinous cancer differs from adenocarcinoma in terms of clinical and histopathological characteristics. Mucinous cancer is a distinct form of colorectal cancer (CRC) found in 10–15% of patients with CRC. Mucinous cancer differs from adenocarcinoma in terms of clinical and histopathological characteristics [1, 2]. Surgery is the main common management in the form of abdominoperineal resection (APR) [3, 4] as neoadjuvant and adjuvant therapy remains undefined for these diseases [4]. In our case, complete resection of the lesion was difficult due to its large size and surrounding tissue infiltration. To achieve this, we performed an abdominoperineal resection with vertical rectus abdominis myocutaneous flap (VRAM) tissue reconstruction.

The aim of reporting this case is to highlight its rarity, clinicopathological characteristics, the treatment provided, management of postoperative sequelae with its final outcome and subsequent follow-up.

## 2. Case Presentation

A 66-year-old gentleman with underlying type II diabetes mellitus was initially treated under the colorectal team for his horseshoe abscess with complex fistula. We performed an examination under anaesthesia with endoanal ultrasound and drainage using modified Hanley procedure in October 2018 which histopathological examination (HPE) of biopsies taken showed adenocarcinoma with mucinous differentiation. We proceeded with a colonoscopy in November 2018 which showed grossly normal bowel mucosa but were unable to complete the scope procedure due to tight angle at hepatic flexure. CT TAP in December showed a perineal mass with minimal extension into left gluteal subcutaneous tissue with tiny lung nodule. MRI of the pelvis showed a large mass at the intergluteal cleft size 6.8 × 5.3 × 6.3 cm (AP × *W* × CC) with high T2-w mucinous content—epicentre in lower anal canal with defect and involvement of the internal and external sphincter as well as suspicious involvement in the posterior aspect of the levator ani/puborectalis sling.

**Figure 1:**
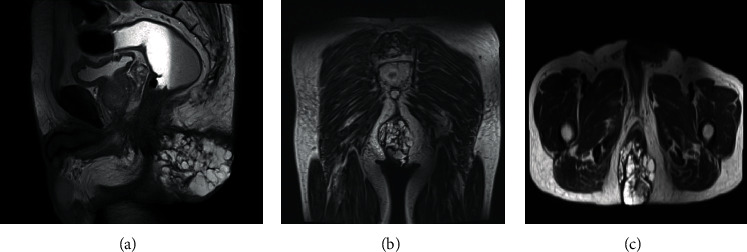
(a–c) MRI of the pelvis showing large mass involving the anal sphincter.

After confirmation of diagnosis, management options were discussed in a multidisciplinary team meeting. In view of extensive involvement of the surrounding soft tissues and anal canal, surgical resection by abdominoperineal resection followed by vertical rectus abdominis myocutaneous flap (VRAM) tissue reconstruction was planned by the colorectal and plastic surgery teams. HPE from the operation confirmed the diagnosis of mucinous adenocarcinoma, well differentiated with no LN involvement (Figure 1).

## 3. Operative Technique

**Figure 2:**
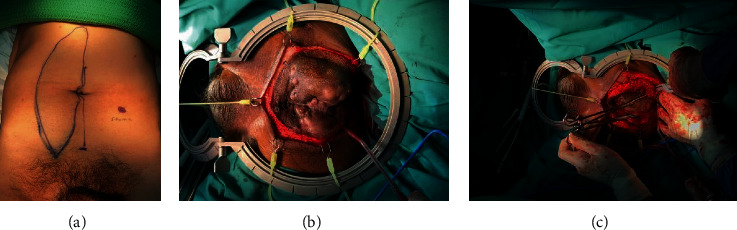
(a–c) Preoperative marking of VRAM flap and en bloc resection.

The colorectal team performed an abdominal perineal resection; the patient was placed in the Lloyd Davis position, and midline laparotomy was performed. Medial and lateral mobilisation of the sigmoid colon was performed followed with proximal mobilization up to splenic flexure and distally to the pelvic flood along with total mesorectal excision. Both inferior mesenteric artery and the vein were ligated in their origin. The sigmoid colon was divided with linear stapler and brought out as an end stoma at the left iliac fossa. Perineal incision was performed with R0 en bloc resection of the tumour along with coccygectomy (Figure 2).

**Figure 3:**
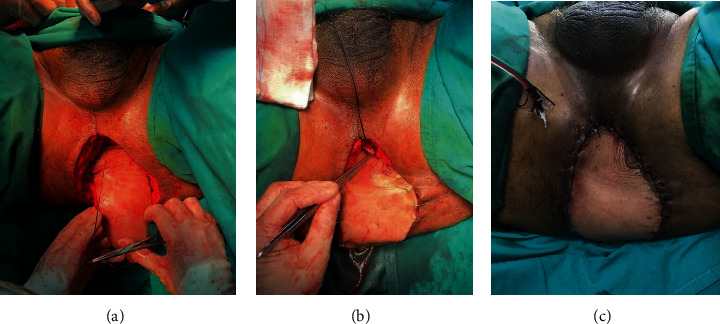
(a–c) Harvested rectus flap tunnelled into the pelvis.

The plastic surgery team continued the operation with their VRAM flap procedure. The rectus abdominis muscle was palpated and marked; an incision was made along the area of interest. The right rectus abdominis muscle was dissected from posterior, lateral, and medial portions of the rectus sheath. Perforator vessels were ligated and divided. The deep inferior epigastric artery and vein were identified. The harvested rectus flap was rotated 180 degree on its deep inferior epigastric artery pedicle and tunnelled via intraperitoneal route into the pelvis. The flap was secured by a multilayered closure using absorbable and nonabsorbable sutures (Figure 3).

Unfortunately, the patient developed intestinal obstruction during his recovery period in the intensive care unit a few days postoperatively. The dilated loops of bowel may have compressed the pedicle leading to the nonviability and subsequent loss of the VRAM flap. Exploratory laparotomy and adhesiolysis was performed by the colorectal team, and debridement of flap tissue was performed by the plastic surgery team. Once confirmed that the excision margins were clear, the perineal wound was managed with serial negative pressure wound therapy. Subsequently, his perineal wound successfully healed by granulation and secondary intention. The patient was discharged well from the hospital.

## 4. Discussion

The acceptable treatment of malignancy originating from a fistula-in-ano is APR of the rectum with wide local excision of the perineal lesions [2–6]. This rather radical surgery is aimed at locoregional control of the disease as the course remains for a long period at the local and regional levels, with persistent perianal sepsis and risk of intestinal obstruction. The common risk factors associated with etiopathogenesis are benign inflammatory conditions like chronic anal fistula, perianal abscess, syphilis diabetes, tuberculosis, and diabetes mellitus. Apart from chronic perianal abscess leading to fistula-in-ano, our patient did not have any other condition such as Crohn's disease. In our patient, we did not perform any radiotherapy and chemotherapy as adenocarcinoma in perianal abscess is not like a rectal disease which responds well to radiotherapy and chemotherapy. The prognosis is not as good as rectal cancer in regard to neoadjuvant therapy. We believe giving any of these modalities will not give any positive outcome to the patient and will only delay the definite surgical management.

For our patient, we performed a combined surgery with the plastic surgery team, in which they had performed a perineal reconstruction to facilitate wound closure and healing. Vertical rectus abdominis musculocutaneous flap was used to close the perineal wound.

**Figure 4:**
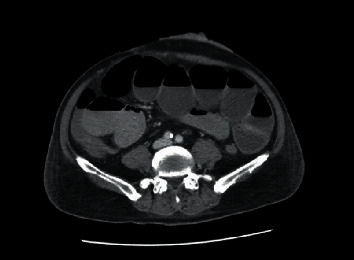
CT of the abdomen showing the small bowel obstruction with transition zone at the proximal ileum.

Unfortunately, he developed intestinal obstruction on day 5 postoperatively, which we noticed from the ceased stoma output and distended abdomen. This intestinal obstruction causing abdominal distension is what we believe led to the compromise of the flap vascularity. CT scan of the abdomen showed that there was a transition zone between the distal small bowel and the terminal ileum (Figure 4). Our team brought him back for another surgery. Interestingly, the cause of the obstruction was found to be by the intestinal herniation between the bowels which is a rare cause of intestinal obstruction. The distended herniated bowel loops most likely compressed the vascular pedicle supplying the flap resulting in the unlikely flap survival. After rearrangement of the bowel, the nonviable flap tissue was debrided. The resulting wound was managed by our dedicated plastic team with advanced wound care, including negative pressure wound therapy (NPWT). The wound is now fully healed.

Some nonsurgical discussions are also worth mentioning such as the role of additional chemotherapy/radiotherapy. This topic has been debated widely. While there is evidence that chemoradiotherapy improves survival, some authors prefer not to add chemoradiotherapy arguing that the prognosis is good after surgery alone if treated early, and that follow-up is sufficient. Others advocate neoadjuvant chemo- and radiotherapy, noting that this increases the median survival for up to 3 years [6]. For this case, our patient had no chemotherapeutic or radiotherapy preoperatively.

Preoperative CT scans of the thorax, abdomen, and pelvis show only local disease with no involvement of the lymph node and no distant metastasis. Although using other modalities such as MRI and endorectal ultrasound also may be considered in this case, these are proven better in tissue differentiation and to asses local invasion into surrounding however endorectal ultrasound was not performed for this patient because its not feasible for the patient.

Mucinous adenocarcinoma (MAC) differs from adenocarcinoma (AC) in so far as it may have originated from the same genetic cause but instead of producing less mucus, produces far more. MAC is characterized by the formation of a tumour comprised of at least 50 percent mucin. Mucin is not mucus per se but rather the glycoprotein component of mucus and other bodily fluids (such as saliva and breast milk). It is this mucinous component that many believe helps a tumour spread more aggressively as it sees beyond the walls of the tumour to the adjacent tissue [7]. Commonly, cancers will spread through the lymphatic and inguinal lymph node which are the most frequent sites of metastasis. In our case where the lesion is in the perianal area, the spreading of cancer may spread to the lateral pelvic lymph node. However, in our patient, he did not have any lymph node involvement.

As such, MAC has long been considered a more aggressive form of AC and to be far less receptive to treatment.

Adenocarcinomas associated with fistula-in-ano are usually of the mucinous (colloid) histological type. Our patient had a well-differentiated histological grade, pT3NX which has a good prognosis but also poor sensitivity to chemotherapy or radiotherapy. Prognosis depends on the size of the tumour, presence of metastases, and the histological grade.

The manifestation of a carcinoma in chronic fistula-in-ano is possibly due to chronic inflammation although the rarity of the condition precludes any definite assumption in regard to the etiologic relationship of the fistula and carcinoma [8]. A rectal carcinoma may present as a fistula, and it may be difficult to determine whether the tumour is a complication of a long-standing perianal fistula or whether the perianal fistula is merely a manifestation of the malignancy itself. In the present case, the initial indication was a horseshoe abscess and complex fistula. Tissue diagnosis from the initial operation showed a mucinous type of adenocarcinoma; hence, we then proceeded with a definitive multidisciplinary treatment.

## 5. Conclusion

Carcinoma is rare complication of horseshoe abscess with complex fistula. It is debatable question that fistula leading to carcinoma or carcinoma leads to fistula formation. In this case, recurrent abscess with chronic fistula-in-ano with repeated perineal sepsis has led to the formation of mucinous adenocarcinoma. So any case of chronic fistula-in-ano and complex fistulas should be subjected for histopathological study before treating, and regular follow-ups are necessary for the operated cases of fistula-in-ano to rule out the conversion of fistula into malignancy. It is necessary, therefore, to evaluate the entire gastrointestinal tract by colonoscopy to rule out any possibility of transformation of malignant cell from proximal gastrointestinal cancer as there is possibility of deposition of malignant cell in the granulation tissue of fistula arising from more proximal cancer.

In regard to squamous carcinoma which responds well to radiotherapy and chemotherapy, it is not applicable to our case as neoadjuvant therapy was debatable, with questionable sensitivity to a mucinous type of adenocarcinoma of perianal region, that is why the decision to proceed with the definite procedure of abdominal perineal resection with a VRAM flap as perineal reconstruction. In addition, due to the rarity of this tumor and the lack of sufficient patients for controlled trials, there is no consensus regarding diagnosis and treatment strategies.
